# Di-μ-oxido-bis­[(4-formyl-2-methoxy­phenolato-κ*O* ^1^)oxido(1,10-phenan­throline-κ^2^ *N*,*N*′)vanadium(V)]

**DOI:** 10.1107/S1600536809032516

**Published:** 2009-08-22

**Authors:** Zhenghua Guo, Lianzhi Li, Tao Xu, Jinghong Li

**Affiliations:** a School of Chemistry and Chemical Engineering, Liaocheng University, Shandong 252059, People’s Republic of China

## Abstract

The title complex, [V_2_(C_8_H_7_O_3_)_2_O_4_(C_12_H_8_N_2_)_2_], is a centrosymmetric dimer formed by two V^V^ complex units bridged by two μ_2_-oxido groups. The V^V^ atom is six-coordinated by three oxide O atoms, one O atom from a vanillinate ligand and two N atoms from a 1,10-phenanthroline ligand in a significantly distorted octa­hedral geometry. In the crystal structure, weak inter­molecular C—H⋯O hydrogen bonds connect the mol­ecules into a three-dimensional network.

## Related literature

For general background to vanadium complexes, see: Dong *et al.* (2000 ▶); Thompson *et al.* (1999 ▶); Yuan *et al.* (2003 ▶). For related structures, see: Li *et al.* (2004 ▶); Mokry & Carrano (1993 ▶).
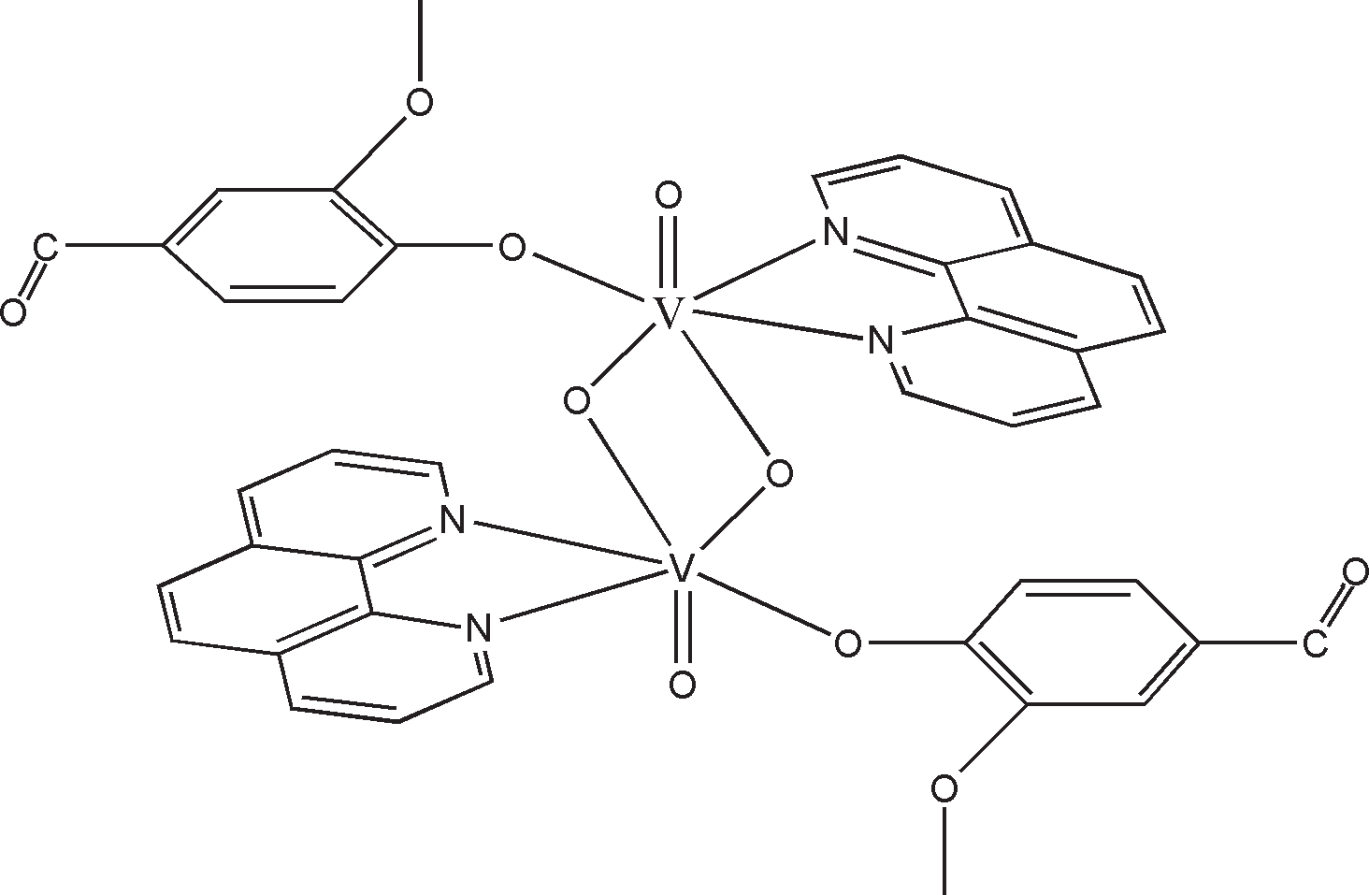


## Experimental

### 

#### Crystal data

[V_2_(C_8_H_7_O_3_)_2_O_4_(C_12_H_8_N_2_)_2_]
*M* *_r_* = 828.56
Triclinic, 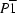

*a* = 9.3453 (18) Å
*b* = 9.786 (2) Å
*c* = 11.090 (3) Å
α = 80.097 (2)°
β = 65.672 (1)°
γ = 71.535 (1)°
*V* = 875.6 (3) Å^3^
*Z* = 1
Mo *K*α radiation
μ = 0.60 mm^−1^
*T* = 298 K
0.21 × 0.18 × 0.17 mm

#### Data collection

Bruker SMART 1000 CCD diffractometer
Absorption correction: multi-scan (*SADABS*; Sheldrick, 1996 ▶) *T*_min_ = 0.884, *T*_max_ = 0.904
4641 measured reflections
3038 independent reflections
2372 reflections with *I* > 2σ(*I*)
*R*_int_ = 0.046

#### Refinement

*R*[*F* ^2^ > 2σ(*F* ^2^)] = 0.061
*wR*(*F* ^2^) = 0.177
*S* = 1.07
3038 reflections
254 parameters
H-atom parameters constrained
Δρ_max_ = 0.84 e Å^−3^
Δρ_min_ = −0.82 e Å^−3^

### 

Data collection: *SMART* (Bruker, 2007 ▶); cell refinement: *SAINT* (Bruker, 2007 ▶); data reduction: *SAINT*; program(s) used to solve structure: *SHELXS97* (Sheldrick, 2008 ▶); program(s) used to refine structure: *SHELXL97* (Sheldrick, 2008 ▶); molecular graphics: *SHELXTL* (Sheldrick, 2008 ▶); software used to prepare material for publication: *SHELXTL*.

## Supplementary Material

Crystal structure: contains datablocks global, I. DOI: 10.1107/S1600536809032516/hy2219sup1.cif

Structure factors: contains datablocks I. DOI: 10.1107/S1600536809032516/hy2219Isup2.hkl

Additional supplementary materials:  crystallographic information; 3D view; checkCIF report

## Figures and Tables

**Table 1:** Selected bond lengths (Å)

V1—O3	1.898 (3)
V1—O4	1.657 (3)
V1—O5	1.610 (3)
V1—O4^i^	2.346 (3)
V1—N1	2.148 (3)
V1—N2	2.245 (3)

Symmetry code: (i) 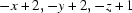.

**Table 2:** Hydrogen-bond geometry (Å, °)

*D*—H⋯*A*	*D*—H	H⋯*A*	*D*⋯*A*	*D*—H⋯*A*
C10—H10⋯O1^ii^	0.93	2.48	3.393 (6)	168
C16—H16⋯O4^iii^	0.93	2.44	3.192 (5)	138
C8—H8*A*⋯O5^iv^	0.96	2.68	3.280 (6)	121
C11—H11⋯O5^v^	0.93	2.67	3.312 (5)	127
C1—H1⋯O5^vi^	0.93	2.62	3.441 (6)	148

Symmetry codes: (ii) 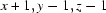; (iii) 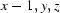; (iv) 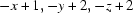; (v) 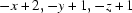; (vi) 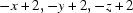.

**Fig. 1.**
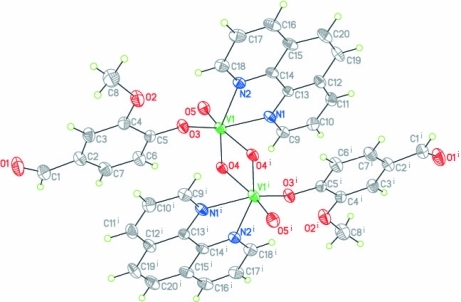
The molecular structure of the title compound, showing 30% probability displacement ellipsoids. [Symmetry code: (i) -x+2, -y+2, -z+1.]

**Fig. 2.**
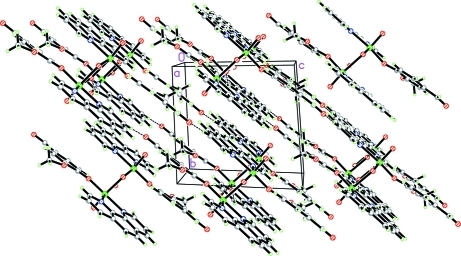
The crystal packing of the title compound with hydrogen bonds (dashed lines).

## supplementary crystallographic information

### Comment

There is an increased interest in vanadium complexes due to their possible uses in pharmaceuticals for the treatment of diabetes (Thompson *et al.*, 1999) and their practical applications in catalysis and material science (Yuan *et al.*, 2003). The vanadium complexes with 1,10-phenanthroline ligand have been reported to exhibit potent antitumor activity (Dong *et al.*, 2000). Vanillin is an useful organic compound with multifunctional groups including aldehyde, ether and phenol. In an effort to uncover the chemistry and biochemistry of vanadium with nitrogen- and oxygen-containing ligands, we report herein the synthesis and crystal structure of a new binuclear vanadium(V) complexes with mixed ligands of vanillin and 1,10-phenanthroline.

The molecular structure of the title complex is shown in Fig.1. In the presence of atmosphere, V^IV^ is oxidized to V^V^. The complex is centrosymmetric dimer formed by two V^V^ complex untis bridged by two µ_2_-oxido groups. The V^V^ atom is six-coordinated by three oxido O atoms, one O atom from a vanillinate ligand and two N atoms from a 1,10-phenanthroline ligand in a significantly distorted octahedral geometry (Table 1). O3, O4, N1 and N2 are situated in the equatorial plane and O5 and O4^i^ [symmetry code: (i) -x+2, -y+2, -z+1] are in the axial positions. The V^V^ atom deviates from the least-squares plane of O3, O4, N1 and N2 by 0.319 (1) Å. In the complex, V1—O4 is 1.657 (3) Å and V1—O4^i^ is 2.346 (3) Å, which illustrates that it is a very asymmetric bridge. The asymmetric structure is similar to that previously reported (Li *et al.*, 2004; Mokry & Carrano, 1993).

There are extensive C—H···O hydrogen bonds in the crystal structure (Table 2). As shown in Fig. 2, the neighboring binuclear complex molecules are connected by the intermolecular hydrogen bonds into a three-dimensional network.

### Experimental

Vanillin (0.152 g, 1 mmol) was dissolved in 5 ml absolute methanol and vanadyl sulfate hydrate (0.225 g, 1 mmol) was added to the solution, which was stirred and refluxed for 2 h at 323 K. Then, a methanol solution (5 ml) of 1,10-phenanthroline (0.198 g, 1 mmol) was added to the solution. The mixture was stirred and refluxed for 3 h at 323 K. The obtained brown solution was cooled to room temperature and filtered. The filtrate was kept at room temperature for 30 d. The crystals suitable for X-ray diffraction were obtained.

### Refinement

H atoms were placed in geometrically calculated positions and allowed to ride on their parent atoms, with C—H = 0.93 (CH) and 0.96 (CH_3_) Å and with *U*_iso_(H) = 1.2(or 1.5 for methyl)*U*_eq_(C).

### Crystal data

**Table:**

[V_2_(C_8_H_7_O_3_)_2_O_4_(C_12_H_8_N_2_)_2_]	*Z* = 1
*M**_r_* = 828.56	*F*(000) = 424
Triclinic, *P*1	*D*_x_ = 1.571 Mg m^−^^3^
Hall symbol: -P 1	Mo *K*α radiation, λ = 0.71073 Å
*a* = 9.3453 (18) Å	Cell parameters from 943 reflections
*b* = 9.786 (2) Å	θ = 2.5–25.8°
*c* = 11.090 (3) Å	µ = 0.60 mm^−^^1^
α = 80.097 (2)°	*T* = 298 K
β = 65.672 (1)°	Needle, colorless
γ = 71.535 (1)°	0.21 × 0.18 × 0.17 mm
*V* = 875.6 (3) Å^3^	

### Data collection

**Table:**

Bruker SMART 1000 CCD diffractometer	3038 independent reflections
Radiation source: fine-focus sealed tube	2372 reflections with *I* > 2σ(*I*)
graphite	*R*_int_ = 0.046
φ and ω scans	θ_max_ = 25.0°, θ_min_ = 2.0°
Absorption correction: multi-scan (*SADABS*; Sheldrick, 1996)	*h* = −10→11
*T*_min_ = 0.884, *T*_max_ = 0.904	*k* = −11→8
4641 measured reflections	*l* = −13→12

### Refinement

**Table:**

Refinement on *F*^2^	Primary atom site location: structure-invariant direct methods
Least-squares matrix: full	Secondary atom site location: difference Fourier map
*R*[*F*^2^ > 2σ(*F*^2^)] = 0.061	Hydrogen site location: inferred from neighbouring sites
*wR*(*F*^2^) = 0.177	H-atom parameters constrained
*S* = 1.07	*w* = 1/[σ^2^(*F*_o_^2^) + (0.1054*P*)^2^ + 0.2207*P*] where *P* = (*F*_o_^2^ + 2*F*_c_^2^)/3
3038 reflections	(Δ/σ)_max_ < 0.001
254 parameters	Δρ_max_ = 0.84 e Å^−^^3^
0 restraints	Δρ_min_ = −0.81 e Å^−^^3^

### Fractional atomic coordinates and isotropic or equivalent isotropic displacement parameters (Å^2^)

**Table:**

	*x*	*y*	*z*	*U*_iso_*/*U*_eq_	
V1	0.93548 (8)	0.89398 (7)	0.61983 (7)	0.0324 (3)	
N1	1.0193 (4)	0.7386 (3)	0.4733 (3)	0.0337 (7)	
N2	0.7109 (4)	0.8658 (3)	0.6109 (3)	0.0329 (7)	
O1	0.6378 (5)	1.4243 (4)	1.1968 (4)	0.0683 (10)	
O2	0.4949 (4)	1.1848 (4)	0.8894 (3)	0.0544 (9)	
O3	0.7773 (3)	1.0430 (3)	0.7319 (3)	0.0385 (7)	
O4	1.1051 (3)	0.9466 (3)	0.5544 (3)	0.0340 (6)	
O5	0.9616 (4)	0.7689 (3)	0.7300 (3)	0.0439 (7)	
C1	0.7557 (7)	1.3424 (6)	1.1248 (5)	0.0544 (12)	
H1	0.8544	1.3312	1.1321	0.065*	
C2	0.7590 (6)	1.2582 (5)	1.0265 (5)	0.0448 (11)	
C3	0.6170 (5)	1.2648 (5)	1.0102 (4)	0.0434 (10)	
H3	0.5168	1.3212	1.0643	0.052*	
C4	0.6250 (5)	1.1877 (5)	0.9139 (4)	0.0366 (9)	
C5	0.7772 (5)	1.1044 (4)	0.8284 (4)	0.0350 (9)	
C6	0.9175 (5)	1.0962 (5)	0.8477 (5)	0.0435 (10)	
H6	1.0182	1.0399	0.7942	0.052*	
C7	0.9070 (6)	1.1723 (5)	0.9469 (5)	0.0465 (11)	
H7	1.0011	1.1654	0.9601	0.056*	
C8	0.3380 (6)	1.2439 (6)	0.9822 (6)	0.0684 (16)	
H8A	0.3289	1.1997	1.0684	0.103*	
H8B	0.2582	1.2264	0.9578	0.103*	
H8C	0.3195	1.3459	0.9840	0.103*	
C9	1.1750 (5)	0.6719 (4)	0.4093 (4)	0.0379 (9)	
H9	1.2535	0.6951	0.4269	0.046*	
C10	1.2269 (5)	0.5683 (4)	0.3167 (4)	0.0423 (10)	
H10	1.3373	0.5232	0.2742	0.051*	
C11	1.1117 (6)	0.5345 (4)	0.2897 (4)	0.0448 (11)	
H11	1.1438	0.4656	0.2287	0.054*	
C12	0.9468 (5)	0.6033 (4)	0.3537 (4)	0.0398 (10)	
C13	0.9066 (5)	0.7047 (4)	0.4456 (4)	0.0301 (8)	
C14	0.7401 (5)	0.7743 (4)	0.5200 (4)	0.0326 (9)	
C15	0.6154 (5)	0.7467 (5)	0.4962 (5)	0.0419 (10)	
C16	0.4555 (5)	0.8190 (5)	0.5727 (5)	0.0503 (12)	
H16	0.3683	0.8040	0.5616	0.060*	
C17	0.4273 (5)	0.9118 (5)	0.6636 (5)	0.0491 (11)	
H17	0.3208	0.9608	0.7142	0.059*	
C18	0.5583 (5)	0.9333 (5)	0.6811 (4)	0.0407 (10)	
H18	0.5375	0.9967	0.7437	0.049*	
C19	0.8183 (6)	0.5772 (5)	0.3311 (5)	0.0510 (12)	
H19	0.8438	0.5120	0.2686	0.061*	
C20	0.6622 (6)	0.6447 (5)	0.3981 (5)	0.0519 (12)	
H20	0.5813	0.6254	0.3809	0.062*	

### Atomic displacement parameters (Å^2^)

**Table:**

	*U*^11^	*U*^22^	*U*^33^	*U*^12^	*U*^13^	*U*^23^
V1	0.0238 (4)	0.0321 (4)	0.0453 (4)	−0.0028 (3)	−0.0170 (3)	−0.0116 (3)
N1	0.0333 (18)	0.0263 (17)	0.0485 (19)	−0.0051 (14)	−0.0234 (15)	−0.0047 (14)
N2	0.0259 (17)	0.0315 (18)	0.0425 (19)	−0.0053 (14)	−0.0152 (14)	−0.0043 (14)
O1	0.067 (3)	0.074 (3)	0.067 (2)	−0.010 (2)	−0.023 (2)	−0.037 (2)
O2	0.0297 (16)	0.074 (2)	0.063 (2)	−0.0057 (15)	−0.0158 (15)	−0.0325 (17)
O3	0.0272 (14)	0.0411 (16)	0.0473 (17)	0.0006 (12)	−0.0148 (12)	−0.0200 (13)
O4	0.0232 (13)	0.0363 (15)	0.0449 (15)	−0.0048 (11)	−0.0131 (12)	−0.0143 (12)
O5	0.0441 (17)	0.0433 (17)	0.0491 (17)	−0.0068 (14)	−0.0247 (14)	−0.0059 (14)
C1	0.061 (3)	0.060 (3)	0.050 (3)	−0.020 (3)	−0.025 (3)	−0.009 (2)
C2	0.050 (3)	0.042 (3)	0.050 (3)	−0.012 (2)	−0.025 (2)	−0.007 (2)
C3	0.038 (2)	0.045 (3)	0.044 (2)	−0.0042 (19)	−0.0143 (19)	−0.013 (2)
C4	0.033 (2)	0.040 (2)	0.037 (2)	−0.0078 (18)	−0.0139 (17)	−0.0046 (18)
C5	0.036 (2)	0.035 (2)	0.033 (2)	−0.0100 (18)	−0.0114 (17)	−0.0038 (17)
C6	0.035 (2)	0.041 (2)	0.057 (3)	0.0017 (18)	−0.025 (2)	−0.014 (2)
C7	0.041 (3)	0.051 (3)	0.058 (3)	−0.010 (2)	−0.028 (2)	−0.007 (2)
C8	0.035 (3)	0.086 (4)	0.079 (4)	−0.006 (3)	−0.013 (3)	−0.033 (3)
C9	0.030 (2)	0.032 (2)	0.052 (2)	−0.0033 (17)	−0.0182 (19)	−0.0039 (18)
C10	0.038 (2)	0.031 (2)	0.049 (2)	0.0012 (18)	−0.013 (2)	−0.0095 (19)
C11	0.058 (3)	0.030 (2)	0.045 (2)	−0.007 (2)	−0.019 (2)	−0.0094 (19)
C12	0.048 (3)	0.030 (2)	0.048 (2)	−0.0098 (19)	−0.026 (2)	−0.0034 (18)
C13	0.030 (2)	0.030 (2)	0.033 (2)	−0.0093 (16)	−0.0142 (16)	−0.0009 (16)
C14	0.028 (2)	0.030 (2)	0.044 (2)	−0.0087 (16)	−0.0190 (17)	0.0004 (17)
C15	0.040 (2)	0.038 (2)	0.063 (3)	−0.0146 (19)	−0.034 (2)	0.006 (2)
C16	0.038 (3)	0.052 (3)	0.076 (3)	−0.018 (2)	−0.037 (2)	0.005 (2)
C17	0.027 (2)	0.052 (3)	0.068 (3)	−0.009 (2)	−0.020 (2)	−0.002 (2)
C18	0.027 (2)	0.040 (2)	0.053 (3)	−0.0036 (18)	−0.0163 (19)	−0.004 (2)
C19	0.060 (3)	0.049 (3)	0.064 (3)	−0.016 (2)	−0.037 (3)	−0.014 (2)
C20	0.057 (3)	0.051 (3)	0.071 (3)	−0.020 (2)	−0.043 (3)	−0.002 (2)

### Geometric parameters (Å, °)

**Table:**

V1—O3	1.898 (3)	C7—H7	0.9300
V1—O4	1.657 (3)	C8—H8A	0.9600
V1—O5	1.610 (3)	C8—H8B	0.9600
V1—O4^i^	2.346 (3)	C8—H8C	0.9600
V1—N1	2.148 (3)	C9—C10	1.398 (6)
V1—N2	2.245 (3)	C9—H9	0.9300
N1—C9	1.324 (5)	C10—C11	1.372 (6)
N1—C13	1.355 (5)	C10—H10	0.9300
N2—C18	1.319 (5)	C11—C12	1.392 (6)
N2—C14	1.350 (5)	C11—H11	0.9300
O1—C1	1.196 (6)	C12—C13	1.400 (6)
O2—C4	1.359 (5)	C12—C19	1.426 (6)
O2—C8	1.403 (6)	C13—C14	1.425 (6)
O3—C5	1.314 (5)	C14—C15	1.407 (5)
O4—V1^i^	2.346 (3)	C15—C16	1.395 (7)
C1—C2	1.459 (6)	C15—C20	1.440 (7)
C1—H1	0.9300	C16—C17	1.362 (7)
C2—C7	1.383 (7)	C16—H16	0.9300
C2—C3	1.393 (6)	C17—C18	1.398 (6)
C3—C4	1.375 (6)	C17—H17	0.9300
C3—H3	0.9300	C18—H18	0.9300
C4—C5	1.416 (6)	C19—C20	1.335 (7)
C5—C6	1.390 (6)	C19—H19	0.9300
C6—C7	1.385 (6)	C20—H20	0.9300
C6—H6	0.9300		
			
O5—V1—O4	105.63 (14)	C6—C7—H7	119.4
O5—V1—O3	99.73 (14)	O2—C8—H8A	109.5
O4—V1—O3	105.13 (13)	O2—C8—H8B	109.5
O5—V1—N1	91.67 (14)	H8A—C8—H8B	109.5
O4—V1—N1	93.98 (13)	O2—C8—H8C	109.5
O3—V1—N1	154.01 (13)	H8A—C8—H8C	109.5
O5—V1—N2	99.66 (13)	H8B—C8—H8C	109.5
O4—V1—N2	152.20 (13)	N1—C9—C10	123.1 (4)
O3—V1—N2	81.37 (12)	N1—C9—H9	118.5
N1—V1—N2	73.69 (12)	C10—C9—H9	118.5
O5—V1—O4^i^	172.60 (13)	C11—C10—C9	118.7 (4)
O4—V1—O4^i^	77.95 (12)	C11—C10—H10	120.6
O3—V1—O4^i^	85.35 (11)	C9—C10—H10	120.6
N1—V1—O4^i^	81.55 (11)	C10—C11—C12	120.0 (4)
N2—V1—O4^i^	75.66 (10)	C10—C11—H11	120.0
C9—N1—C13	117.7 (3)	C12—C11—H11	120.0
C9—N1—V1	123.9 (3)	C11—C12—C13	117.1 (4)
C13—N1—V1	118.3 (3)	C11—C12—C19	124.3 (4)
C18—N2—C14	118.8 (3)	C13—C12—C19	118.6 (4)
C18—N2—V1	126.4 (3)	N1—C13—C12	123.4 (4)
C14—N2—V1	114.8 (2)	N1—C13—C14	116.2 (3)
C4—O2—C8	118.0 (4)	C12—C13—C14	120.4 (3)
C5—O3—V1	132.1 (3)	N2—C14—C15	123.2 (4)
V1—O4—V1^i^	102.05 (12)	N2—C14—C13	117.0 (3)
O1—C1—C2	126.1 (5)	C15—C14—C13	119.9 (4)
O1—C1—H1	117.0	C16—C15—C14	116.6 (4)
C2—C1—H1	117.0	C16—C15—C20	125.5 (4)
C7—C2—C3	119.6 (4)	C14—C15—C20	117.9 (4)
C7—C2—C1	119.1 (4)	C17—C16—C15	119.8 (4)
C3—C2—C1	121.3 (4)	C17—C16—H16	120.1
C4—C3—C2	119.9 (4)	C15—C16—H16	120.1
C4—C3—H3	120.1	C16—C17—C18	120.0 (4)
C2—C3—H3	120.1	C16—C17—H17	120.0
O2—C4—C3	125.1 (4)	C18—C17—H17	120.0
O2—C4—C5	114.2 (4)	N2—C18—C17	121.6 (4)
C3—C4—C5	120.7 (4)	N2—C18—H18	119.2
O3—C5—C6	123.8 (4)	C17—C18—H18	119.2
O3—C5—C4	117.4 (4)	C20—C19—C12	121.2 (4)
C6—C5—C4	118.8 (4)	C20—C19—H19	119.4
C7—C6—C5	119.8 (4)	C12—C19—H19	119.4
C7—C6—H6	120.1	C19—C20—C15	121.9 (4)
C5—C6—H6	120.1	C19—C20—H20	119.1
C2—C7—C6	121.2 (4)	C15—C20—H20	119.1
C2—C7—H7	119.4		
			
O5—V1—N1—C9	−77.6 (3)	C3—C4—C5—C6	3.1 (6)
O4—V1—N1—C9	28.2 (3)	O3—C5—C6—C7	175.7 (4)
O3—V1—N1—C9	166.0 (3)	C4—C5—C6—C7	−1.8 (6)
N2—V1—N1—C9	−177.1 (3)	C3—C2—C7—C6	2.3 (7)
O4^i^—V1—N1—C9	105.4 (3)	C1—C2—C7—C6	−176.7 (4)
O5—V1—N1—C13	101.4 (3)	C5—C6—C7—C2	−0.9 (7)
O4—V1—N1—C13	−152.8 (3)	C13—N1—C9—C10	−0.7 (6)
O3—V1—N1—C13	−15.1 (5)	V1—N1—C9—C10	178.3 (3)
N2—V1—N1—C13	1.8 (3)	N1—C9—C10—C11	0.5 (6)
O4^i^—V1—N1—C13	−75.6 (3)	C9—C10—C11—C12	0.2 (6)
O5—V1—N2—C18	91.8 (3)	C10—C11—C12—C13	−0.7 (6)
O4—V1—N2—C18	−112.8 (4)	C10—C11—C12—C19	179.1 (4)
O3—V1—N2—C18	−6.6 (3)	C9—N1—C13—C12	0.1 (6)
N1—V1—N2—C18	−179.2 (4)	V1—N1—C13—C12	−178.9 (3)
O4^i^—V1—N2—C18	−94.0 (3)	C9—N1—C13—C14	177.6 (3)
O5—V1—N2—C14	−90.9 (3)	V1—N1—C13—C14	−1.4 (4)
O4—V1—N2—C14	64.4 (4)	C11—C12—C13—N1	0.5 (6)
O3—V1—N2—C14	170.6 (3)	C19—C12—C13—N1	−179.2 (4)
N1—V1—N2—C14	−2.0 (3)	C11—C12—C13—C14	−176.8 (4)
O4^i^—V1—N2—C14	83.2 (3)	C19—C12—C13—C14	3.4 (6)
O5—V1—O3—C5	55.9 (3)	C18—N2—C14—C15	−0.1 (6)
O4—V1—O3—C5	−53.3 (3)	V1—N2—C14—C15	−177.6 (3)
N1—V1—O3—C5	170.7 (3)	C18—N2—C14—C13	179.5 (3)
N2—V1—O3—C5	154.3 (3)	V1—N2—C14—C13	2.0 (4)
O4^i^—V1—O3—C5	−129.5 (3)	N1—C13—C14—N2	−0.5 (5)
O5—V1—O4—V1^i^	173.33 (13)	C12—C13—C14—N2	177.1 (3)
O3—V1—O4—V1^i^	−81.74 (13)	N1—C13—C14—C15	179.1 (3)
N1—V1—O4—V1^i^	80.47 (12)	C12—C13—C14—C15	−3.3 (6)
N2—V1—O4—V1^i^	18.6 (3)	N2—C14—C15—C16	−0.3 (6)
O4^i^—V1—O4—V1^i^	0.0	C13—C14—C15—C16	−179.9 (4)
O1—C1—C2—C7	177.4 (5)	N2—C14—C15—C20	−178.9 (4)
O1—C1—C2—C3	−1.6 (8)	C13—C14—C15—C20	1.5 (6)
C7—C2—C3—C4	−1.0 (7)	C14—C15—C16—C17	0.6 (6)
C1—C2—C3—C4	178.0 (4)	C20—C15—C16—C17	179.2 (4)
C8—O2—C4—C3	−11.6 (7)	C15—C16—C17—C18	−0.6 (7)
C8—O2—C4—C5	169.6 (4)	C14—N2—C18—C17	0.2 (6)
C2—C3—C4—O2	179.5 (4)	V1—N2—C18—C17	177.3 (3)
C2—C3—C4—C5	−1.7 (6)	C16—C17—C18—N2	0.2 (7)
V1—O3—C5—C6	17.7 (6)	C11—C12—C19—C20	178.5 (4)
V1—O3—C5—C4	−164.7 (3)	C13—C12—C19—C20	−1.7 (7)
O2—C4—C5—O3	4.3 (5)	C12—C19—C20—C15	−0.1 (8)
C3—C4—C5—O3	−174.6 (3)	C16—C15—C20—C19	−178.3 (5)
O2—C4—C5—C6	−178.0 (4)	C14—C15—C20—C19	0.2 (7)

Symmetry codes: (i) −*x*+2, −*y*+2, −*z*+1.

### Hydrogen-bond geometry (Å, °)

**Table:**

*D*—H···*A*	*D*—H	H···*A*	*D*···*A*	*D*—H···*A*
C10—H10···O1^ii^	0.93	2.48	3.393 (6)	168
C16—H16···O4^iii^	0.93	2.44	3.192 (5)	138
C8—H8A···O5^iv^	0.96	2.68	3.280 (6)	121
C11—H11···O5^v^	0.93	2.67	3.312 (5)	127
C1—H1···O5^vi^	0.93	2.62	3.441 (6)	148

Symmetry codes: (ii) *x*+1, *y*−1, *z*−1; (iii) *x*−1, *y*, *z*; (iv) −*x*+1, −*y*+2, −*z*+2; (v) −*x*+2, −*y*+1, −*z*+1; (vi) −*x*+2, −*y*+2, −*z*+2.
